# Co-occurrence of Smoking and Unhealthy Diet in the Brazilian Adult Population

**DOI:** 10.5935/abc.20190222

**Published:** 2019-10

**Authors:** Priscila Maria Stolses Bergamo Francisco, Daniela de Assumpção, Deborah Carvalho Malta

**Affiliations:** 1 Universidade Estadual de Campinas - Saúde Coletiva, Campinas, SP - Brazil; 2 Universidade Federal de Minas Gerais - Escola de Enfermagem, Belo Horizonte, MG - Brazil

**Keywords:** Tobacco Use Disorders, Feeding, Risk Factors, Risk reduction Behavior, Chronic Disease, Adult Health, Health Status Disparities

## Abstract

**Background:**

Smoking and an inadequate diet are behavioral risk factors that contribute to the majority of deaths and disabilities caused by noncommunicable diseases.

**Objectives:**

To estimate the prevalence of the co-occurrence of smoking and inadequate diet and identify associated factors in adults.

**Methods:**

A cross-sectional population-based study was conducted with a sample of 28,950 Brazilian adults (18 to 59 years old). Data were obtained from Sistema de Vigilância por Inquérito Telefônico (Vigitel [Brazilian Health Surveillance Telephone Survey]) in 2014. Independent associations were investigated using Poisson hierarchical regression analysis with 5% significance level.

**Results:**

The prevalence of the co-occurrence of smoking and unhealthy eating was 8.6% (95% CI: 7.9-9.3) and was higher among individuals residing in the southern region of the country than in those living in the central western region (PR = 1.50; 95% CI: 1.18-1.89), those with no private health insurance (PR = 1.14; 95% CI: 1.03-1.25), those who drank alcohol abusively (binge drinkers) (PR = 3.22; 95% CI: 2.70-3.85) and those who self-rated their health as fair (PR = 1.65; 95% CI: 1.36-1.99) or poor/very poor (PR = 1.70; 95% CI: 1.18-2.44). The prevalence of both factors was lower among individuals residing in the northeastern region of the country, women, individuals with brown skin color, those with a spouse, the more educated ones and those with overweight or obesity.

**Conclusion:**

The more vulnerable segments to the co-occurrence of the risk factors studied were men residing in the southern region of the country, individuals with a lower socioeconomic status and those who reported binge drinking. Interventions addressing multiple behavioral risk factors adapted to specific contexts could have a greater impact on the Brazilian population.

## Introduction

Behavioral risk factors are responsible for the majority of deaths due to noncommunicable diseases (NCDs)^1,2^ and part of the diseases resulting from these conditions.^3-5^ Such factors included smoking, abusive alcohol intake, inadequate diet, physical inactivity, obesity, dyslipidemia, excessive animal fat intake and insufficient intake of fruits and vegetables.^6,7^ According to a study conducted in 52 countries, these factors, combined with arterial hypertension, diabetes mellitus and psychosocial stress account for 90% and 94% of the attributable risk of cardiovascular disease among men and women, respectively.^8^

The World Health Organization attributes smoking to an estimated six million deaths per year. Insufficient intake of fruits and vegetables corresponds annually to 2.7 million deaths, 31% of ischemic heart diseases, 11% of cerebrovascular diseases and 19% of gastrointestinal cancers in the world.^9^ Despite the reduction in the percentage of smokers in Brazil in recent years,^10,11^ population-based health surveys have indicated that the prevalence of smoking is greater among adults (40 to 59 years old) and those with a lower education level.^10-12^ Moreover, the prevalence of an unhealthy diet is high,^10,13,14^ especially among men, adolescents and individuals with a lower education level.^13,14^

NCDs have multiple causes that occur simultaneously, resulting in distinct effects.^5^ Studies indicate that the accumulation of two or more modifiable risk factors increases the occurrence of NCDs^8,15,16^ and cardiovascular diseases^8^ and is related to the overall death rate as well as death due to specific causes.^1,2^ Risk behaviors are harmful actions that either increase the probability of disease or impede the recovery of health.^17^ Therefore, behavioral (modifiable) risk factors are component causes that contribute to increased morbidity and mortality rates due to cardiovascular diseases, diabetes mellitus and cancer in adults and seniors.^1,5,15^ The greatest impact of exposure to these factors is seen at more advanced ages. However, early signs of changes in health status occur more frequently from 40 years of age.^18,19^

Brazil has an adult population (18 to 59 years of age) of approximately 114 million. The co-occurrence of smoking and an unhealthy diet has been under-investigated in the literature. Exposure to behavioral risk factors begins early in life^16,18^ and is consolidated in adulthood,^13^ with effects on health in different phases of life. Therefore, the objective of this study was to estimate the co-occurrence of smoking and unhealthy eating practices in the Brazilian adult population as well as determine associations with socio-demographic characteristics and health indicators.

## Methods

A cross-sectional population-based study was conducted with a sample of adults (18 to 59 years old) residing in the capitals of the 26 states of Brazil and the Federal District. The data were extracted from the records of 28,950 individuals interviewed in *Sistema de Vigilância por Inquérito Telefônico* (Vigitel [Brazilian Health Surveillance Telephone Survey]), in 2014.

**Figure 1:**
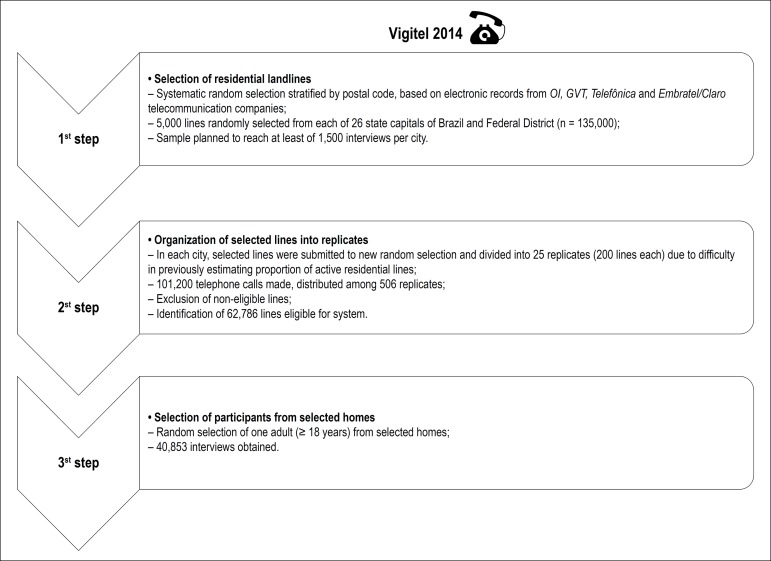
Flowchart of sample selection process. Vigitel, 2014.

A minimum sample of 1,500 individuals in each city was established to estimate the frequency of any risk factor in the adult population^20^ considering a 95% confidence interval and a maximum error of three percentage points.^7^ Data collection was performed in three steps. The first step consisted of systematic random selection of at least five thousand telephone lines. This systematic selection stratified by postal code was performed using records of residential landlines registered with telephone companies. The lines selected in each city were submitted to a second random selection divided into replicates of 200 lines, with each replicate reproducing the same proportion of lines per postal code of the original registry. The third step was the random selection of one of the adults residing in the selected homes (after identification) among the lines considered eligible for the system. The following were excluded in this step: business lines, out-of-service or nonexistent lines and lines for which there was no answer after six calls on different days and at different times, including weekends and evening hours^7^ (Figure 1).

Weighting factors were used to compensate for the bias of the non-universal coverage of landlines. Using a post-stratification weight calculated based on 36 analysis categories by sex (female and male), age group (18-24, 25-34, 35-44, 45-54, 55-64 and ≥65 years old) and level of education (none or incomplete primary school, complete primary school or incomplete high school, complete high school or incomplete university degree and complete university degree), the estimates were adjusted to the population. The “rake” method was used for the calculation of the post-stratification weight of each individual in the sample. Information on the sample design of the Vigitel survey, the data collection instruments and procedures used in the interviews is published elsewhere.^7^

In the present study, the co-occurrence of smoking and an inadequate diet was considered the variable of interest. A smoker was considered any individual who answered affirmatively to the following question: “Do you currently smoke?,” irrespective of the number of cigarettes, frequency and duration. The indicator of an unhealthy diet was created from a set of foods that serve as markers of the intake profile associated with protection from chronic diseases (beans, fruits, milk, raw vegetables and cooked vegetables) and risk for chronic diseases (red meat, sweets and sweetened beverages). Scores ranging from zero to four were attributed depending on the food and intake frequency. Markers of the protection category ingested daily and those of the risk category never or rarely ingested were not scored (zero). Maximum of four points was attributed to protective foods never or rarely consumed and risk foods ingested daily (Table 1). The total score was determined by the sum of food items and ranged from 0 to 32 points, with higher scores indicating poorer dietary quality. This variable was then categorized considering distribution terciles. Individuals in the 2^nd^ and 3^rd^ terciles (14 or more points) were grouped together, creating a dichotomous variable for unhealthy eating (yes or no). Co-occurrence was determined by the simultaneous occurrence of both of these conditions (smoking and unhealthy diet).

**Table 1:** Scoring scale for unhealthy consumption of foods. Vigitel, 2014

Foods	0	1	2	3	4
Beans	Every day	5 to 6 days a week	3 to 4 days a week	1 to 2 days a week	Never or hardly ever
Fruits	Every day	5 to 6 days a week	3 to 4 days a week	1 to 2 days a week	Never or hardly ever
Raw vegetables^1^	Every day	5 to 6 days a week	3 to 4 days a week	1 to 2 days a week	Never or hardly ever
Cooked vegetables^2^	Every day	5 to 6 days a week	3 to 4 days a week	1 to 2 days a week	Never or hardly ever
Milk	Every day	5 to 6 days a week	3 to 4 days a week	1 to 2 days a week	Never or hardly ever
Red meat^3^	Never or hardly ever	1 to 2 days a week	3 to 4 days a week	5 to 6 days a week	Every day
Sweetened soft drink or artificial juice	Never orhardly ever	1 to 2 days a week	3 to 4 days a week	5 to 6 days a week	Every day
Sweets^4^	Never or hardly ever	1 to 2 days a week	3 to 4 days a week	5 to 6 days a week	Every day

1 Lettuce and tomato salad or salad with any other raw vegetable.

2 Consumption of vegetables cooked with food or in soup, such as collards, carrot, eggplant, zucchini, except potato, cassava or yam.

3 Red meat: beef, pork, goat

4 Consumption of sweets, such as ice cream, chocolate, cakes, cookies, etc.

The following socio-demographic variables were considered: macro-region of the country (North, Northeast, Central West, South and Southeast), sex (male and female), age group (18 to 39 and ≥ 40 years old), skin color/ethnicity (white, black, yellow, brown and indigenous), marital status (with and without a spouse), education (0 to 8, 9 to 11 and 12 or more years of study) and having a private health insurance plan (yes or no). The following variables related to behavior and health status were considered: body mass index (BMI) (< 25 kg/m^2^, ≥ 25 to < 30 kg/m^2^ and ≥ 30 kg/m^2^), binge drinking [five or more drinks for men and four or more drinks for women on a single occasion in the previous 30 days (yes or no)], practice of physical activity (active or inactive) and self-rated health (very good/good, fair or poor/very poor). Weight and height were self-reported by the respondents. BMI was calculated for all records based on the imputation of the measures of weight and height using the “hot deck” method.^7^ The following diseases were also considered: arterial hypertension, diabetes mellitus and dyslipidemia (all categorized as “yes or no”).

### Statistical analysis

Descriptive analysis was performed for the characterization of the study population. Age (continuous variable) was expressed as mean and respective 95% confidence interval. Categorical variables were expressed as relative frequency (percentage).

Prevalence values were estimated for smoking, unhealthy eating and the co-occurrence of both of these variables of interest according to socio-demographic characteristics, other behavioral factors and health conditions. Associations were determined between the co-occurrence of the risk factors and variables selected using Pearson’s chi-square test with second-order correction (Rao & Scott), considering a 5% significance level. Next, prevalence ratios were estimated and adjusted for sex and age according to socio-demographic characteristics, behavioral factors and health conditions. A hierarchical Poisson regression model was used considering two sets of variables: 1) socio-demographic and 2) behavioral/health conditions. The variables from the first block were incorporated into the model. Those that remained significant after adjustments by the other variables on the same hierarchical level remained in the model, to which the second block of variables was incorporated. All variables with p-value < 0.05 after adjustments for variables on the same and higher hierarchical level remained in the final model. The analyses were performed using the Stata statistical package, version 12.0.

The objectives of the survey were made clear to all individuals contacted by telephone and written consent was substituted with verbal consent. The Vigitel study received approval from the National Human Research Ethics Committee of the Brazilian Health Ministry (certificate number: 355.590, June 26, 2013).

## Results

Mean age of the sample was 36.4 years (95% CI: 36.1-36.6); the majority was women (53.0%) and young adults (59.4%). The prevalence of the co-occurrence of the risk factors was 8.6% (95% CI: 7.9-9.3).

Table 2 shows the prevalence of smoking and an inadequate diet as well as associations with the other variables. No associations were found between smoking and BMI, physical inactivity, hypertension, diabetes or dyslipidemia (p > 0.05). All variables were associated with unhealthy eating, except for having a private health insurance plan (p = 0.102) and BMI (p = 0.196).

**Table 2:** Prevalence of smoking and unhealthy diet in adults according to geographic region, socio-demographic characteristics, behavioral factors and health conditions. Vigitel, Brazil, 2014

Variable/categories	n	(%)	Smoking	p*	Unhealthy diet	p*
**Geographic region**						
Central West	4,068	11.8	9.9	< 0.001	70.3	0.005
Northeast	9,912	25.7	7.8		68.4	
North	8,267	10.6	7.8		72.6	
Southeast	4,110	44.1	13.3		69.8	
South	2,593	7.8	15.6		72.2	
**Sex**						
Male	11,704	47.0	13.2	< 0.001	76.6	< 0.001
Female	17,246	53.0	9.2		64.2	
**Age group (in years)**						
18 to 39	13,960	59.4	10.2	0.005	75.3	< 0.001
40 to 59	14,990	40.6	12.4		62.4	
**Skin color/ethnicity**						
White	10,780	41.9	11.9	< 0.001	69.6	
Black	2,713	11.7	12.8		70.1	
Yellow	790	3.0	9.0		82.0	0.002
Brown	12,015	41.8	9.1		69.9	
Indigenous	411	1.6	16.8		72.6	
**Marital status**						
With spouse	13,565	50.7	12.1	0.012	73.2	< 0.001
Without spouse	15,052	49.3	10.1		67.0	
**Education (in years)**						
0 to 8	5,720	29.7	16.3	< 0.001	66.4	< 0.001
9 to 11	12,325	42.0	10.5		71.6	
12 or more	10,905	28.3	6.6		71.2	
**Private health insurance**						
No	13,923	51.9	13.6	< 0.001	70.9	0.102
Yes	14,954	48.1	8.5		69.1	
**BMI (kg/m^2^)**						
< 25	13,651	48.9	11.9	0.136	70.8	0.196
≥ 25 to < 30	10,122	33.9	10.4		68.7	
≥ 30	5,177	17.2	10.3		70.1	
**Physical inactivity**						
No	25,363	87.6	11.1	0.908	69.2	< 0.001
Yes	3,587	12.4	11.0		75.7	
**Binge drinking**						
No	24,048	81.5	8.0	< 0.001	67.8	< 0.001
Yes	4,902	18.5	2.5		79.3	
**Self-rated health**						
Very good/good	19,214	67.4	9.5		69.2	
Fair	8,328	28.5	13.4	< 0.001	71.8	0.028
Poor/very poor	1,193	4.1	18.7		73.8	
**Arterial hypertension**						
No	22,887	81.7	11.1	0.743	71.1	< 0.001
Yes	6,063	18.3	11.4		65.2	
**Diabetes mellitus**						
No	27,356	94.9	11.1	0.811	70.9	< 0.001
Yes	1,594	5.1	11.4		53.3	
**Dyslipidemia**						
No	22,835	83.0	11.1	0.630	70.6	0.029
Yes	6,115	17.0	10.4		67.6	

* p-value of chi-square test with Rao-Scott correction. BMI: body mass index

In the simple analysis, differences were found with regard to the region of the country, sex, skin color/ethnicity, education and health insurance (p < 0.05). Associations were found between the co-occurrence of risk factors and both binge drinking (p < 0.001) and self-rated health (p < 0.001). Higher frequencies of the co-occurrence of risk factors were found among individuals residing in the southern and southeastern regions of the country compared to those residing in the central western region. Higher frequencies were also found among individuals who did not have private health insurance, binge drinkers and individuals who reported their health to be fair, poor or very poor at the time of the interview. After controlling for sex and age, the prevalence of the co-occurrence of risk factors was lower among individuals who resided in the northern and northeastern regions of the country, women, individuals with brown skin color, those with a spouse and those with excess weight. The prevalence of the two risk factors also reduced significantly with higher education levels (p < 0.001) (Table 3).

**Table 3:** Prevalence and crude and adjusted prevalence ratios of smoking and inadequate diet according to geographic region, socio-demographic characteristics, behavioral factors and health conditions. Vigitel, Brazil, 2014

Variables/categories	n	Smoking/Inadequate diet	PR_crude_ (95% CI)	PR*_adjusted_ (95% CI)
**Geographic region**		< 0.001		
Central West	3,929	8.0	1	1
Northeast	9,200	5.8	0.73 (0.59-0.89)	0.73 (0.60-0.90)
North	7,791	6.2	0.78 (0.61-0.99)	0.78 (0.62-0.99)
Southeast	3,878	10.3	1.29 (1.04-1.59)	1.28 (1.03-1.57)
South	2,481	12.2	1.53 (1.23-1.90)	1.51 (1.21-1.87)
**Sex**		< 0.001		
Male	10,983	10.8	1	1
Female	16,296	6.7	0.61 (0.52-0.72)	0.60 (0.51-0.71)
**Age group (in years)**		0.167		
18 to 39	12,985	8.2	1	1
40 to 59	14,294	9.2	1.12 (0.95-1.31)	1.15 (0.98-1.34)
**Skin color/ethnicity**		0.008		
White	10,263	9.5	1	1
Black	2,534	9.3	0.98 (0.76-1.25)	0.98 (0.76-1.25)
Yellow	733	6.8	0.71 (0.44-1.16)	0.75 (0.46-1.22)
Brown	11,283	7.2	0.75 (0.61-0.92)	0.76 (0.62­-0.93)
Indigenous	389	13.3	1.40 (0.84-2.32)	1.38 (0.85-2.24)
**Marital status**		0.062		
Without spouse	12,621	9.3	1	1
With spouse	14,351	8.0	0.85 (0.72-1.01)	0.72 (0.60-0.87)
**Education (in years)**		< 0.001		
0 to 8	5,227	12.4	1	1
9 to 11	11,526	8.4	0.67 (0.56-0.81)	0.70 (0.58-0.85)
12 or more	10,526	5.3	0.42 (0.33-0.53)	0.45 (0.35-0.56)
**Private health insurance**		< 0.001		
Yes	14,324	6.7	1	1
No	12,886	10.5	1.25 (1.15-1.36)	1.24 (1.14-1.35)
**BMI(kg/m^2^)**		0.055		
<25	12,867	9.5	1	1
≥25 to < 30	9,564	7.8	0.82 (0.68-0.98)	0.73 (0.61-0.89)
≥30	4,848	7.9	0.83 (0.66-1.03)	0.75 (0.60-0.94)
**Physical inactivity**		0.993		
No	23,992	8.6	1	1
Yes	3,287	8.6	1.00 (0.78-1.28)	0.97 (0.75-1.25)
**Binge drinking**		< 0.001		
No	22,644	6.0	1	1
Yes	4,635	19.9	3.30 (2.81-3.88)	3.17 (2.68-3.76)
**Self-rated health**		< 0.001		
Very good/good	18,312	7.3	1	1
Fair	730	11.1	1.53 (1.28-1.82)	1.57 (1.31-1.87)
Poor/very poor	1,048	13.1	1.80 (1.31-2.48)	1.91 (1.40-2.61)
**Arterial hypertension**		0.815		
No	21,559	8.6	1	1
Yes	5,720	8.4	0.97 (0.79-1.19)	0.88 (0.71-1.08)
**Diabetes mellitus**		0.658		
No	25,780	8.6	1	1
Yes	1,499	8.0	0.92 (0.63-1.33)	0.83 (0.57-1.21)
**Dyslipidemia**		0.475		
No	21,485	8.7	1	1
Yes	5,794	8.1	0.93 (0.75-1.13)	0.87 (0.70-1.06)

n: number of individuals in the unweighted sample.

* PR_adjusted_: prevalence ratio adjusted for sex and age. 95% CI: 95% confidence interval.

Table 4 shows the hierarchical Poisson regression model of factors associated with the co-occurrence of smoking and unhealthy eating. The prevalence of both factors was lower in the northeastern region of the country (PR = 0.67; 95% CI: 0.54-0.83) and higher in the southern region (PR = 1.40; 95% CI: 1.11-1.77) compared to the central western region. The prevalence was approximately 40% lower among women and was also lower among individuals with brown skin and those who lived with a spouse. The prevalence reduced significantly with higher education levels and was approximately 16% higher among individuals without private health insurance (PR = 1.16; 95% CI: 1.05-1.27) after controlling for region of residence and other socio-demographic factors. Regarding behaviors and health conditions, excess weight was inversely associated with the co-occurrence of both risk factors. The prevalence was higher among those who considered their health to be fair or poor/very poor. Moreover, a strong, independent, statistically significant association was found between binge drinking and the co-occurrence of the risk factors considered (PR = 3.22; 95% CI: 2.70-3.85).

**Table 4:** Hierarchical Poisson regression model for factors associated with co-occurrence of smoking and inadequate diet in Brazilian adults. Vigitel, Brazil, 2014

Variables/categories	PR^a^_adjusted_ (95% CI)	PR^b^_adjusted_ (95% CI)
**Geographic region**		
Central West	1	1
Northeast	0.67 (0.54-0.83)	0.68 (0.55-0.84)
North	0.78 (0.61-1.00)	0.84 (0.65-1.08)
Southeast	1.24 (1.00-1.55)	1.30 (1.05-1.61)
South	1.40 (1.11-1.77)	1.50 (1.18-1.89)
**Sex**		
Male	1	1
Female	0.63 (0.53-0.75)	0.77 (0.65-0.92)
**Skin color/ethnicity**		
White	1	1
Black	0.89 (0.69-1.15)	0.84 (0.65-1.09)
Yellow	0.72 (0.42-1.21)	0.71 (0.42-1.18)
Brown	0.73 (0.59-0.89)	0.70 (0.57-0.85)
Indigenous	1.26 (0.78-2.05)	1.40 (0.87-2.26)
**Marital status**		
Without spouse	1	1
With spouse	0.78 (0.65-0.93)	0.85 (0.71-1.02)
**Education (in years)**		
0 to 8	1	1
9 to 11	0.62 (0.51-0.76)	0.60 (0.49-0.72)
12 or more	0.41 (0.32-0.53)	0.39 (0.30-0.51)
**Private health insurance**		
Yes	1	1
No	1.16 (1.05-1.27)	1.14 (1.03-1.25)
**Body mass index (kg/m^2^)**		
< 25		1
≥ 25 to < 30		0.73 (0.60-0.89)
≥ 30		0.76 (0.60-0.97)
**Binge drinking**		
No		1
Yes		3.22 (2.70-3.85)
**Self-rated health**		
Very good/good		
Fair		1.65 (1.36-1.99)
Poor/very poor		1.70 (1.18-2.44)

95% CI: 95% confidence interval; PR: prevalence ratio.

a adjusted for geographic region and socio-demographic characteristics;

b adjusted for geographic region, socio-demographic characteristics, behavioral factors and health conditions.

## Discussion

The prevalence of the co-occurrence of smoking and an unhealthy diet was 8.6%. In a study conducted in England with a population aged 16 to 64 years, the prevalence of the co-occurrence of smoking and inadequate diet (measured by the consumption of beans, vegetables and fruits) was 25.5% among men and 23.6% among women.^21^ In this study, which considered four lifestyle risk factors, the prevalence of smoking was 28.0% and insufficient intake of beans, fruits and vegetables was represented by the failure to have five portions of these foods, as recommended. The divergence in frequencies may be explained by cultural diversity and the diversity of eating habits in different populations. In a study conducted in the city of Botucatu (state of São Paulo, Brazil), Berto et al.^22^ investigated the association between smoking and other behavioral risk factors in adults and found the co-occurrence of smoking and low intake of fresh fruits and vegetables (12.9% and 12.3% among men and 5.8% and 5.1% among women, respectively). In a study conducted with adults in the city of Florianópolis (state of Santa Catarina), the prevalence of the co-occurrence of smoking and an inadequate diet was 3.5%; inadequate diet was considered the reported intake of fruits and vegetables ≤5 days per week.^13^

In an American study with an adult population (≥20 years), smokers had a diet of poor quality, with less intake of fruits, vegetables, dairy products and whole grains as well as a greater percentage of energy from solid fats, alcohol and added sugar.^23^ A study conducted with adult smokers found that fruits and vegetables, noncaffeinated beverages, sweets and dairy products worsened the sensory attributes of cigarettes, whereas meats, alcoholic beverages and caffeinated drinks improved the sensory attributes.^24^ Haibach et al.^25^ found that smokers who ingested more fruits and vegetables had lower levels of nicotine dependence and greater occurrence of smoking cessation.

To make the dietary indicator employed in the present study, we considered whole milk a marker of healthy diet. The results of epidemiological studies evaluating dietary sources of saturated fat have revealed the lack of an association or beneficial effects of dairy products on cardiovascular diseases.^26,27^ Considering the gaps in the scientific literature on the association between milk consumption and health, Lamarche et al.^27^ report the need to investigate whether whole milk and skim milk have different effects on health. Data from the 2008-2009 Brazilian Family Budget Survey revealed that the adult population has low consumption of dairy products (100 g/ml per day),^28^ but high consumption of red and processed meats (90 g per day), with more than 80% of the participants exceeding the limit recommended by the World Cancer Research Fund (300 g per week).^29^

It should be stressed that the Vigitel survey did not address the consumption of processed meats or quantify the consumption of red meat. Therefore, only the frequency of weekly consumption was considered in the present study, regardless of the presence of visible fat. A previous study evaluating the attributable fraction of cancer in the adult population due to different exposures found that red meat is a risk factor for colon and rectal cancer when ingested at a rate of more than 70 g/day.^30^

In the present investigation, the co-occurrence of risk factors was higher in the southern region of Brazil and lower in the northeastern region than in the central western region. Reduced prevalence of smoking was found in all regions of the country between 2006 and 2013, but with the highest rates found in the South and the lowest in the Northeast,^31^ which may partially explain the present findings regarding the co-occurrence of smoking and an inadequate diet. Moreover, according to the 2008-2009 Family Budget Survey, northeastern and southern Brazil have different profiles in terms food purchase, with greater availability of red meat, processed meats, bacon, soft drinks and alcoholic beverages in the southern region.^32^ Regional disparities in the distribution of modifiable risk factors are found in Brazil.^7,13,22,31^ In the United States, a study addressing the co-occurrence of five healthy behaviors (not smoking, regular practice of physical activity, not consuming alcohol, maintaining one’s weight and sleeping the recommended number of hours) in the adult population (≥21 years old) found geographic variations in the percentage distribution adjusted for age for the number of grouped factors.^33^

Regarding other socio-demographic characteristics, studies indicate that the prevalence of multiple risk factors is higher among young adults, men, individuals with a lower socioeconomic status (lower income and education) and those who live alone.^13,21,34^ In the present study, the reduced co-occurrence of risk factors was found with higher education levels. Studies report an association between higher education level and healthy behaviors/health conditions.^10-14,19,21,34^ The prevalence of co-occurrence was also lower among those with self-declared brown skin. In the city of Florianópolis (state of Santa Catarina), Silva et al.^13^ found a greater occurrence of the accumulation of four risk factors in black adults. In the USA, racial/ethnic differences were found for five behaviors considered.^33^ A study analyzing differences in the prevalence of risk factors for chronic diseases according to ethnicity/skin color found that, compared to whites, brown individuals smoked less and consumed fewer fruits, soft drinks and sweets as well as more beans, whole milk and meat with visible fat.^35^ No studies were found in the national or international literature on the co-occurrence of smoking and an inadequate diet according to skin color/ethnicity.

The prevalence of the co-occurrence of risk factors was higher among individuals without a private health insurance plan. A study involving Brazilian adults found that individuals with private health insurance smoked less, ate better and practiced more physical activity during leisure hours.^36^ The Health Ministry has taken several actions for reducing inequalities in the access and offer of healthcare services. The National Health Promotion Policy defends integral care, considering health promotion to be a strategy for organizing the actions and services of the public healthcare system, with a focus on factors that determine the health-disease process, intersectoral actions, social participation and the construction of healthy environments on individual and collective levels.^37^ The National Food and Nutrition Policy and the Dietary Guide for the Brazilian Population^38^ are important support instruments for the promotion of healthy eating within the Brazilian public health system (SUS, in its Portuguese acronym).

The prevalence of the co-occurrence of smoking and inadequate diet was lower among adults with excess weight. The inverse association found after adjustment for socio-demographic characteristics, behavioral factors and health conditions may be partially explained by the fact that smoking exerts an influence on metabolic processes; smokers weigh, on average, 4 kg less than non-smokers due to the increase in the metabolic rate, concomitantly with suppression of appetite.^39^

The prevalence of the co-occurrence of risk factors was higher among adults who reported binge drinking, which is a subgroup with greater vulnerability to NCDs. The planning of disease prevention actions should integrate population-based strategies and strategies directed at high-risk subgroups, as both are necessary and work in a synergistic way.^17^ In the epidemiology of chronic diseases, the effect of a risk factor depends on the status of the individual for another factor (present/absent). Thus, the presence of two or more modifiable risk factors potentiates the occurrence of NCDs^8,15,16^ and shorter time to the emergence of a disease leads to reduced healthy life expectancy. Data from four cohort studies on smoking, physical inactivity and obesity among individuals aged 50 to 75 in European countries revealed the impact of the co-occurrence of behavioral risk factors on the reduction in the expectancy of a healthy life free of chronic diseases.^40^

In the present study, the co-occurrence of risk factors was higher among those who did not rate their health positively. The literature describes the association between smoking and a poor perception of health in the adult population.^34,41^ A study involving the Brazilian population ≥18 years of age also found poor assessments of health among individuals who did not consume fruits and vegetables regularly.^41^ A study conducted in Madrid, Spain with 16,043 adults (18 to 64 years old) found that the accumulation of risk factors increased the frequency of perceived poor health in a progressive manner.^34^

No associations were found between the co-occurrence of risk factors and arterial hypertension, diabetes or dyslipidemia. In the analysis of individual risk factors, these conditions were only associated with an inadequate diet. National studies have not found an association between current smoking and arterial hypertension or diabetes, as found for former smokers.^42,43^ A significant reduction in smoking occurred between 2006 and 2015^31,44^ and a substantial increase in excess weight has occurred as a result of negative changes in the eating patterns of the population.^32,45^ It should be stressed that the greatest incidence of these diseases and other health-related problems is found at more advanced ages. In the present study, the co-occurrence of the two risk factors did not necessarily express an additional risk for these outcomes in the adult population analyzed.

Estimates of the clustering of behavioral risk factors for NCDs performed in international studies^1,21,34,40^ have led to the recognition that many of these factors are interrelated.^17^ Effective prevention resides in reducing the concomitant occurrence of various risk factors related to these diseases, on both the individual and collective levels. The incidence of a given disease is important to primary prevention, as the risk is low for the majority of individuals, regardless of the disease.^15^ Strategies on the population level seek to control determinants of the disease with interventions directed at environmental factors that make the disease prevalent.^46^ Stratification of the population according to risk enables the identification of its distribution in the population and the adoption of specific prevention practices focused on priority subgroups.

Interventions that address multiple risk factors can have a greater impact than those focused on isolated behaviors.^2,15^ The co-occurrence of health-related behaviors suggests complementary and substitutive relations. In Brazil, the goal of the Strategic Action Plan to Combat Non-Communicable Diseases is the reduction in the prevalence of smoking in the adult population from 15.1% (2011) to 9.1% (2022). Regarding dietary practices, the goal is to reduce mean salt intake from 12 g (2010) to 5 g (2022). The increase in the consumption of fruits and vegetables is on the list of monitoring indicators of the World Health Organization, but is not on the list of goals.^47^ Global strategies adopted in specific contexts need to be implemented, broadened and, especially, maintained.

The present study has limitations that should be considered. The sample was restricted to the population with a landline at home, which diminished the participation of the northern and northeastern regions of the country, where coverage rates are lower. However, the use of weighting factors minimized the difference between populations with and without a telephone line.^7^ Further limitations include the use of self-reported information and the impossibility of establishing causal relations due to the cross-sectional design of the study. It is not possible to affirm whether individuals with excess weight quit smoking and made changes in eating practices or whether smoking and a poor diet led to weight loss.

## Conclusion

In the present study, the segments that are more vulnerable to the co-occurrence of smoking and an inadequate diet were residents of the southern region of the country, men, individuals with a lower socioeconomic status and those who reported binge drinking. Interventions addressing multiple behavioral risk factors, adapted to specific contexts, could have a greater impact on the Brazilian population. Regarding the management of healthcare services, information obtained from indicators can help guide the implementation, monitoring and assessment of healthcare models and actions directed at health promotion, as well as disease prevention and control.

Considering the increasing social inequality in Brazil and the consolidation of a dietary system centered on monocultures for the production of ultraprocessed foods that are disseminated throughout all social strata of the population through strong marketing strategies, the promotion of health and prevention of worsening of adverse health conditions are powerful and absolutely necessary strategies for reducing the impact of social profile on health and the access of the population to healthy aging.

## References

[1] Gopinath B, Flood VM, Burlutsky G, Mitchell P (2010). Combined influence of health behaviours on total and cause-specific mortality. Arch Intern Med.

[2] Kvaavik E, Batty GD, Ursin G, Huxley R, Gale CR (2010). Influence of individual and combined health behaviours on total and cause-specific mortality in men and women: the United Kingdom health and lifestyle survey. Arch Intern Med.

[3] Schmidt MI, Duncan BB, Azevedo e Silva G, Menezes AM, Monteiro CA, Barreto SM (2011). Chronic non communicable diseases in Brazil: burden and current challenges. Lancet.

[4] Malta DC, Silva JB (2012). Policies to promote physical activity in Brazil. Lancet.

[5] Forouzanfar MH, Alexander L, Anderson HR, Bachman VF, Biryukov S, GBD 2013 Risk Factors Collaborators (2015). Global, regional, and national comparative risk assessment of 79 behavioural, environmental and occupational, and metabolic risks or clusters of risks in 188 countries, 1990-2013: a systematic analysis for the Global Burden of Disease Study 2013. Lancet.

[6] World Health Organization (2014). Noncommunicable diseases country profiles 2014.

[7] Brasil, Ministério da Saúde, Secretaria de Vigilância em Saúde, Departamento de Vigilância de Doenças e Agravos não Transmissíveis e Promoção da Saúde (2015). Vigitel Brasil 2014.

[8] Yusuf S, Hawken S, Ounpuu S, Dans T, Avezum A, Lanas F (2004). Effect of potentially modifiable risk factors associated with myocardial infarction in 52 countries (the INTERHEART study): case-control study. Lancet.

[9] World Health Organization (2011). Global status report on noncommunicable diseases 2010.

[10] Malta DC, Andrade SSCA, Stopa SR, Pereira CA, Szwarcwald CL, Silva Jr JB (2015). Brazilian lifestyles: National Health Survey results, 2013. Epidemiol Serv Saúde.

[11] Malta DC, Vieira ML, Szwarcwald CL, Caixeta R, Brito SMF, Reis AAC (2015). Smoking trends among brazilian population - national household survey, 2008 and the National Health Survey, 2013. Rev Bras Epidemiol.

[12] Silva GA, Valente JG, Malta DC (2011). Trends in smoking among the adult population in Brazilian capitals: a data analysis of telephone surveys from 2006 to 2009. Rev Bras Epidemiol.

[13] Silva DA, Peres KG, Boing AF, González-Chica DA, Peres MA (2013). Clustering of risk behaviors for chronic noncommunicable diseases: a population-based study in southern Brazil. Prev Med.

[14] Claro RM, Santos MAS, Oliveira TP, Pereira CA, Szwarcwald CL, Malta DC (2015). Unhealthy food consumption related to chronic non-communicable diseases in Brazil: National Health Survey, 2013. Epidemiol Serv Saúde.

[15] Goldstein MG, Whitlock EP, DePue J, Planning Committee of the Addressing Multiple Behavioral Risk Factors in Primary Care Project (2004). Multiple behavioral risk factor interventions in primary care. Summary of research evidence. Am J Prev Med.

[16] Dumith SC, Muniz LC, Tassitano RM, Hallal PC, Menezes AM (2012). Clustering of risk factors for chronic diseases among adolescents from Southern Brazil. Prev Med.

[17] Spring B, Moller AC, Coons MJ (2012). Multiple health behaviours: overview and implications. J Public Health.

[18] Barreto SM, Passos VMA, Giatti L (2009). Healthy behavior among Brazilian young adults. Rev Saúde Pública.

[19] Barros MBA, Francisco PMSB, Zanchetta LM, Cesar CLG (2011). Trends in social and demographic inequalities in the prevalence of chronic diseases in Brazil: PNAD: 2003- 2008. Ciên & Saúde Colet.

[20] WHO (1991). Sample size determination in health studies: a practical manual.

[21] Poortinga W (2007). The prevalence and clusterin of four major lifestyle risk factors in an English adult population. Prev Med.

[22] Berto SJP, Carvalhaes MABL, Moura EC (2010). Smoking associated with other behavioral risk factors for chronic non-communicable diseases. Cad Saúde Pública.

[23] Guenther PM, Reedy J, Krebs-Smith SM, Reeve BB (2008). Evaluation of the Healthy Eating Index-2005. J Am Diet Assoc.

[24] McClernon FJ, Westman EC, Rose JE, Lutz AM (2007). The effects of foods, beverages, and other factors on cigarette palatability. Nicotine Tob Res.

[25] Haibach JP, Homish GG, Giovino GA (2013). A longitudinal evaluation of fruit and vegetable consumption and cigarette smoking. Nicotine Tob Res.

[26] Siri-Tarino PW, Chiu S, Bergeron N, Krauss RM (2015). Saturated fats versus polyunsaturated fats versus carbohydrates for cardiovascular disease prevention and treatment. Annu Rev Nutr.

[27] Lamarche B, Givens DI, Soedamah-Muthu S, Krauss RM, Jakobsen MU, Bischoff-Ferrari HA (2016). Does milk consumption contribute to cardiometabolic health and overall diet quality. Can J Cardiol.

[28] Araujo MC, Bezerra IN, Barbosa FS, Junger WL, Yokoo EM, Pereira RA (2013). Macronutrient consumption and inadequate micronutrient intake in adults. Rev Saúde Pública.

[29] Carvalho AM, Selem SS, Miranda AM, Marchioni DM (2016). Excessive red and processed meat intake: relations with health and environment in Brazil. Br J Nutr.

[30] Azevedo e Silva G, de Moura L, Curado MP, Gomes FS, Otero U, Rezende LF (2016). The fraction of cancer attributable to ways of life, infections, occupation, and environmental agents in Brazil in 2020. PLoS One.

[31] Malta DC, Oliveira TP, Luz M, Stopa SR, Silva Junior JB, Reis AAC (2015). Smoking trend indicators in Brazilian capitals, 2006-2013. Ciên & Saúde Colet.

[32] Levy RB, Claro RM, Mondini L, Sichieri R, Monteiro CA (2012). Regional and socioeconomic distribution of household food availability in Brazil, in 2008-2009. Rev Saúde Pública.

[33] Liu Y, Croft JB, Wheaton AG, Kanny D, Cunningham TJ, Lu H (2016). Clustering of five health-related behaviors for chronic disease prevention among adults, United States, 2013. Prev Chronic Dis.

[34] Galán I, Rodriguez-Artalejo F, Tobías A, Díez-Gañán L, Gandarilhas A, Zorrilla B (2005). Clustering of behavioural risk factors and their association with subjective health. Gac Sanit.

[35] Malta DC, Moura L, Bernal RTI (2015). Differentials in risk factors for chronic non-communicable diseases from the race/color standpoint. Ciênc Saúde Coletiva.

[36] Malta DC, Bernal RTI (2014). Comparison of risk and protective factors for chronic diseases in the population with and without health insurance in the Brazilian capitals, 2011. Rev Bras Epidemiol.

[37] Brasil, Ministério da Saúde, Secretaria de Vigilância em Saúde, Secretaria de Atenção à Saúde (2010). Política Nacional de Promoção da Saúde.

[38] Brasil, Ministério da Saúde, Secretaria de Atenção à Saúde, Departamento de Atenção Básica (2014). Guia Alimentar para a População Brasileira.

[39] Chiolero A, Faeh D, Paccaud F, Cornuz J (2008). Consequences of smoking for body weight, body fat distribution, and insulin resistance. Am J Clin Nut.

[40] Stenholm S, Head J, Kivimäki M, Kawachi I, Aalto V, Zins M (2016). Smoking, physical inactivity and obesity as predictors of healthy and disease-free life expectancy between ages 50 and 75: a multicohort study. Int J Epidemiol.

[41] Barros MBA, Zanchetta LM, Moura EC, Malta DC (2009). Auto-evaluación de la salud
y factores asociados, Brasil, 2006. Rev Saúde Pública.

[42] Malta DC, Bernal RTI, Andrade SSCA, Silva MMA, Velasquez-Melendez G (2017). Prevalence of and factors associated with self-reported high blood pressure in Brazilian adults. Rev Saúde Pública.

[43] Malta DC, Bernal RTI, Iser BPM, Szwarcwald CL, Duncan BB, Schmidt MI (2017). Factors associated with self-reported diabetes according to the 2013 National Health Survey. Rev Saúde Pública.

[44] Malta DC, Silva MMA, Moura L, Morais Neto OL (2017). The implantation of the Surveillance System for Non-communicable Diseases in Brazil, 2003 to 2015: successes and challenges. Rev Bras Epidemiol.

[45] Malta DC, Santos MAS, Andrade SSCA, Oliveira TP, Stopa SR, Oliveira MM (2016). Time trend in adult obesity indicators in Brazilian state capitals, 2006-2013. Ciên & Saúde Colet.

[46] Goeffrey Rose (1985). Individuos enfermos y poblaciones enfermas. Int J Epidemiol.

[47] Malta DC, Silva Jr JB (2013). Brazilian Strategic Action Plan to Combat Chronic Non-communicable Diseases and the global targets set to confront these diseases by 2025: a review. Epidemiol Serv Saúde.

